# *circSATB1* Modulates Cell Senescence in Age-Related Acute Myeloid Leukemia: A Mechanistic Proposal

**DOI:** 10.3390/cells14151181

**Published:** 2025-07-31

**Authors:** Linxiang Han, Xi Wen, Ling Zhang, Xingcheng Yang, Ziyan Wei, Haodong Wu, Yichen Zhan, Huiting Wang, Yu Fang

**Affiliations:** 1 Department of Clinical Medicine, School of Medical, Wuhan University of Science and Technology, Wuhan 430065, China; hanlinxiang@wust.edu.cn (L.H.); yangxingcheng@wust.edu.cn (X.Y.); weiziyan@wust.edu.cn (Z.W.); wuhandong@wust.edu.cn (H.W.); yichenzhan@wust.edu.cn (Y.Z.); wanghuiting@wust.edu.cn (H.W.); 2 Department of Environmental Hygiene and Occupational Medicine, School of Public Health, Wuhan University of Science and Technology, Wuhan 430065, China; 202353703442@wust.edu.cn (X.W.); zhangling@wust.edu.cn (L.Z.); 3 Hubei Province Key Laboratory of Occupational Hazard Identification and Control, Wuhan University of Science and Technology, Wuhan 430065, China

**Keywords:** age-related diseases, AML, *circSATB1*, cellular senescence, therapeutic targets

## Abstract

Acute myeloid leukemia (AML) is a malignant hematological tumor with a high prevalence in elderly people, and circular RNA (circRNA) plays an important role in age-related diseases. Induction of cancer cell senescence is a highly promising therapeutic strategy; however, the presence of senescence-associated circRNAs in AML remains to be elucidated. Here, we show that the expression patterns of circRNAs differed between elderly AML patients and healthy volunteers. *circSATB1* was significantly overexpressed in elderly patients and AML cells. Knockdown of *circSATB1* resulted in the inhibition of proliferation and arrest of the cell cycle in the G0/G1 phase; no effect on apoptosis or DNA integrity was observed, and precocious cellular senescence was promoted, characterized by no change in telomere length. Database analysis revealed that there may be two miRNA and nine RNA-binding proteins (RBPs) involved in regulating the cellular functions of *circSATB1*. Our observations uncover *circSATB1*-orchestrated cell senescence in AML, which provides clues for finding more modest therapeutic targets for AML.

## 1. Introduction

AML, the most common type of acute leukemia in adults, is becoming increasingly prominent in terms of incidence and severity against the backdrop of global aging [1]. By 2050, the global population aged 65 and older is projected to increase from 761 million in 2021 to 1.6 billion, rising from 9% to 16% of the total population [2]. The growth rate of the population aged 80 and above is even faster, with an estimated 140 million in 2021 and 459 million (4.5% of the total population) by 2050 [3]. Most AML patients are aged 50 or older, with the highest incidence and mortality observed in those aged 70 and above [4]. From 2010 to 2017, the overall survival rate for AML patients over 70 was only 5% [5]. The incidence of AML exhibits a pronounced age-dependent pattern. According to the SEER (Surveillance, Epidemiology, and End Results) database, the median age at AML diagnosis is 67 years, with 50–60% of patients diagnosed at ≥65 years and over 30% at ≥75 years [6]. As global aging intensifies (the proportion of the population aged 60 and above is expected to reach 22% by 2050), the absolute number of elderly AML patients will continue to rise [7]. Older AML patients typically present with higher genetic risk (e.g., *TP53* mutations and complex karyotype) and poorer performance status (PS ≥ 2 in over 40% of cases) [8], leading to lower treatment tolerance, reduced remission rates, and shorter survival. The complete remission (CR) rate for standard induction chemotherapy in patients ≥65 years is only 30–50%, with a 5-year overall survival (OS) rate of less than 10% [9], significantly lower than the 40–60% observed in younger patients.

CircRNA, a class of highly stable non-coding RNA with multidimensional regulatory functions, exhibits complex regulatory networks in the pathogenesis of age-related diseases and the aging process [10]. Following ischemic stroke, *circSCMH1* enhances vascular endothelial cell repair and neurological recovery by modulating the ubiquitination of fat and obesity-associated (FTO) protein, reducing m6A modification of the lipid phosphatase phospholipid phosphatase 3 (Plpp3) and increasing its mRNA stability. In Alzheimer’s disease (AD) patients [10], *circPSEN1* is significantly up-regulated, promoting amyloid-β (Aβ) production by inhibiting *miR-137* expression and activating the nuclear factor of activated T cells 1 (NFATC1) and epidermal growth factor receptor (EGFR) pathways [11]. *circSTX12* competitively binds to Casitas B-lineage lymphoma (CBL) protein, inhibiting the Hippo/YAP (Yes-associated protein) pathway, promoting adipogenic differentiation of bone marrow mesenchymal stem cells (BMSCs) and suppressing osteogenic differentiation, thereby contributing to bone loss in senile osteoporosis (OP). Antisense oligonucleotides (ASOs) targeting *circSTX12* significantly improve bone microstructure in mouse models [12]. These studies highlight the central regulatory role of circRNA in age-related and aging-associated diseases, providing novel insights for targeted interventions.

Cellular senescence, first proposed by Hayflick in 1961, describes a state of irreversible growth arrest in actively proliferating cells [13]. Cellular senescence represents the third major tumor-suppressive mechanism following apoptosis and DNA damage repair. Senescent cells exhibit irreversible cell cycle arrest while retaining metabolic activity and secrete a variety of factors, including cytokines, chemokines, and extracellular matrix proteases, which collectively constitute the senescence-associated secretory phenotype (SASP). Therapy-induced senescence (TIS), characterized by irreversible cell cycle arrest and activation of immune clearance mechanisms, has emerged as a cutting-edge strategy in cancer treatment, particularly for elderly patients intolerant to chemotherapy or radiotherapy. Senescence-like phenotype mediated by IFI16 in glioblastoma (GBM) suppresses ferroptosis via Heme Oxygenase-1 (HMOX1) activation, conferring radiation resistance. The classic antidiabetic drug glyburide disrupts the binding of Interferon Gamma Inducible Protein 16 (IFI16) to transcription factors, restoring radio sensitivity [12]. Additionally, circRNA-induced tumor cell senescence is gaining attention. Inhibition of *circPETH-147aa* significantly increases Reactive Oxygen Species (ROS) accumulation, inducing DNA damage and cellular senescence [14]. ASOs targeting *circITGB6* markedly suppress tumor progression in colorectal cancer liver metastasis models and induce a senescent phenotype [12]. However, research on circRNA-induced cellular senescence in AML remains limited.

In this study, we identified *circSATB1* as a significantly overexpressed circRNA in AML through whole-transcriptome sequencing and bioinformatics analysis. We validated its circular nature and confirmed its predominant cytoplasmic localization. Further siRNA knockdown experiments demonstrated that *circSATB1* inhibition suppresses cell proliferation and promotes cellular senescence, with no significant effects on apoptosis or DNA damage. These findings suggest that *circSATB1* holds considerable therapeutic potential in AML, possibly serving as a critical therapeutic target for inducing AML cell senescence.

## 2. Materials and Methods

### 2.1. Sample Collection and Transcriptome Sequencing

Discovery Cohort (RNA sequencing): Peripheral blood was collected from 6 AML patients and 3 healthy controls. Demographic and clinical characteristics have been comprehensively documented in Supplementary Material Table S1. Nuclear cells isolated via density gradient centrifugation underwent RNA sequencing (Illumina platform, Novogene, Sacramento, CA, USA). Differentially expressed circRNAs were identified through integrated computational analysis (CIRCexplorer2, CircFinder) with cross-database annotation. Differentially expressed genes (DEGs) were defined as |logFC| > 1 and *p*-value < 0.05. Validation Cohort (Clinical validation): *circSATB1* expression was assessed in 44 independent anticoagulated blood samples (22 AML vs. 22 controls). All procedures complied with institutional ethics guidelines, with written informed consent obtained from participants.

### 2.2. Cell Culture and Treatment

KG-1a, THP-1, and Kasumi cells were purchased from the cell bank of Type Culture Collection of the Chinese Academy of Sciences (Shanghai, China). Cells were maintained in RPMI-1640 medium (HyClone, South Logan, UT, USA) supplemented with fetal bovine serum (FBS; Gibco, Carlabad, CA, USA, 10% for KG-1a and THP-1, 20% for Kasumi) and 1% penicillin/streptomycin (Gibco, Carlabad, CA, USA) under standard conditions (37 °C/5% CO_2_) with 2–3 times weekly passaging. siRNA transfection utilized Zlipo2000 (ZomanBio, Beijing, China) in 6-well plates (4–6 h incubation), followed by 24–72 h culture post-medium replacement. For siRNA transfection, Pepmute (ZomanBio, Beijing, China) complexes (40 pmol siRNA, RT incubation: 5 + 15 min) were applied to plates, with cells harvested after 24–72 h.

Nuclear-cytoplasmic fractionation was performed in THP-1 and Kasumi cells using pre-chilled Cell Fractionation Buffer (Thermo Fisher Scientific, Waltham, MA, USA) supplemented with PMSF (Beyotime, Shanghai, China, 1 mM final concentration). Sequential centrifugations (500× *g*, 4 °C, 5 min) isolated cellular components, followed by lysis in 2× Lysis Binding Solution (Thermo Fisher Scientific, Waltham, MA, USA; β-mercaptoethanol-supplemented to 1% (*v*/*v*)) and ethanol precipitation. RNA was purified via filter cartridge washes (Wash Solutions 1–3) and eluted in 95 °C Elution Buffer. Nuclear/cytoplasmic proteins were reserved for immunoblotting, while RNA underwent reverse transcription for qPCR analysis.

### 2.3. PCR and qPCR

Total RNA was reverse-transcribed using a Hifair^®^ II 1st Strand cDNA Synthesis Kit (gDNA digester plus) (Yeasen, Shanghai, China) according to the manufacturer’s instructions. Briefly, 1 μg total RNA was mixed with gDNA digester and incubated at 42 °C for 30 min, and 85 °C for 5 min. For PCR, 2× Hieff^TM^ PCR Master Mix (With Dye) (Yeasen, Shanghai, China) was used. The cDNA and gDNA PCR products were evaluated using 2% agarose gel electrophoresis. The qPCR was conducted using 2× SYBR Green qPCR Master Mix (Bimake, Houston, TX, USA). GAPDH, β-actin, and U1 were used as controls. For quantification of gene expression, the 2^−∆∆CT^ method was used, and the data were normalized to an endogenous control.

### 2.4. Western Blot Analysis

Cells were lysed in RIPA buffer (Beyotime, Shanghai, China), denatured in 5× loading buffer (100 °C, 10 min), and resolved via SDS-PAGE (10% or 15%). Proteins were transferred to PVDF membranes, blocked with 5% BSA (Biosharp, Hefei, China), and incubated overnight at 4 °C with primary antibodies: anti-GAPDH, Histone H3, CDK2, hTERT, BAX, PCNA and BCL-2 (Abclonal, Wuhan, China). Membranes were washed (TBST × 3), incubated with HRP-conjugated secondary antibodies (1:5000, 1 h, RT), and visualized using a ChemiDoc system (Bio-Rad, Hercules, CA, USA) with ECL (Beyotime Biotechnology, Shanghai, China). Band intensity was quantified via ImageJ (NIH, ImageJ 1.x).

### 2.5. Cell Proliferation

THP-1 cells were incubated with EdU (RiboBio, Guangzhou, China) in complete medium (1:1000 dilution) for 2 h post-transfection 48 h. Cells (1 × 104/well, 96-well plate, n = 3) were fixed with 4% paraformaldehyde (Biosharp, Hefei, China), permeabilized (0.5% Triton X-100), and stained with Apollo reaction mix and Hoechst 33,342 (Beyotime, Shanghai, China; RT, dark, 30 min). Fluorescence imaging was performed using Optoelectronic Technology microscope (Mingmei, Guangzhou, China). Additionally, cell viability was assessed by a CCK-8 assay kit (Biosharp, Hefei, China) in Kasumi cells.

### 2.6. Cell Apoptosis

THP-1 cells were harvested by centrifugation (500× *g*, 5 min) post-transfection 48 h, washed twice with ice-cold PBS, and stained with Annexin V-FITC (Beyotime, Shanghai, China) in binding buffer (4 °C, dark, 15 min), followed by PI addition (4 °C, dark, 5 min). Unstained, FITC-only, and PI-only controls were included. Apoptotic subpopulations (Annexin V-FITC+/PI−: early apoptosis; Annexin V-FITC+/PI+: late apoptosis/necrosis) were quantified via flow cytometry (BD Biosciense, San Jose, CA, USA).

### 2.7. Cell Cycle

THP-1 cells were harvested by centrifugation (500× *g*, 5 min, RT), washed twice with ice-cold PBS, and fixed in 70% ethanol (−20 °C, ≥2 h). After rehydration, pellets were stained with PI (BD Biosciences, San Jose, CA, USA, 50 μg/mL), RNase A (Sigma-Aldrich, St. Louis, MO, USA, 100 μg/mL), and 0.1% Triton X-100 in PBS (Servicebio, Wuhan, China, 37 °C, 30 min, dark). Cell suspensions were filtered through 40 μm mesh and analyzed on a flow cytometer (488 nm laser; PI detection: bandpass filter centered at 585 nm with 40 nm bandwidth [585/40 nm]), with ≥10,000 events recorded per sample. Instrument calibration utilized unstained controls and fluorescent beads.

### 2.8. Comet Assay

The alkaline single-cell gel electrophoresis technique (comet assay) was used to measure the DNA damage in THP-1 cells using an OxiSelect Comet Assay Kit (Cell Biolabs, Chicago, IL, USA). Briefly, liquid agarose was pipetted onto a comet slide and chilled. Next, cell samples were combined with comet agarose, added on top of the base layer, lysed to form nucleoids containing supercoiled loops, and then immersed in alkaline solution. Then, the samples were electrophoresed for 30 min under voltage 25 V and current 300 mA to separate intact DNA from damaged fragments, stained with a diluent DNA dye (Cell Biolabs, Chicago, IL, USA), and visualized by epifluorescence microscopy using a FITC filter (MshOt, Guangzhou, China, 40×). Comet Assay Software Project (CaspLab-1.2.2) was used to quantify the tail parameter.

### 2.9. β-Galactosidase Staining

β-Galactosidase Staining was measured in THP-1 using the β-galactosidase staining kit (BeiboBio, Shanghai, China) according to the manufacturer’s protocol. Harvest cells by centrifugation at 300× *g* for 5 min at room temperature into a 1.5 mL centrifuge tube. Wash once with PBS, add 1 mL of β-galactosidase staining fixative, and fix at room temperature for 15 min. During fixation, slowly shake the tube on a shaker to avoid the cells from clumping together. After centrifugation (300× *g*, 5 min, RT), remove the cell fixative, wash the cells 3 times with PBS, each time for 3 min. Centrifuge (300× *g*, 5 min, RT), remove the PBS, and add 0.5–1 mL of staining working solution to each tube. Incubate at 37 °C overnight. Drop some of the stained cells onto a slide or into a 6-well plate, and observe under a common optical microscope.

### 2.10. Southern Blot

The measurements of terminal restriction fragment (TRF) length were applied using the TeloTAGGG telomere length assay kit (Roche, Basel, Switzerland). Briefly, DNA extraction from KG-1a cells was performed through sequential lysis, and nucleic acid purity was verified by NanoDrop 2000 spectrophotometry (Thermo Fisher, Waltham, MA, USA), confirming the A260/280 ratio (≥1.8). Equal volumes of HinfI and RsaI were combined for concurrent DNA digestion and positive control preparation, while the molecular marker was formulated by mixing 4 μL Bottle 6, 12 μL RNase-free water, and 4 μL Bottle 7. Electrophoresis was conducted in 0.8% agarose gel with 1× TAE running buffer (Servicebio, Wuhan, China) at 50 V for 5 h, followed by sequential post-electrophoretic processing: membrane transfer via capillary action (ambient temperature, overnight), UV cross-linking, prehybridization for 1 h and hybridization (42 °C, 3 h) in freshly prepared buffer, and chemiluminescent detection using Bottle 15 substrate.

### 2.11. Statistical Analysis

Statistical analyses were performed using GraphPad Prism software (version 7.0). Each experiment was independently repeated three times, and the representative data are shown. All the values are presented as mean ± SD of three biologically independent samples. Statistical analyses were performed using a two-way analysis of variance (ANOVA) when comparing at least three groups. The sample size is indicated in the corresponding figure legends. Statistical significance was defined as * *p* < 0.05, ** *p* < 0.01.

## 3. Results

Expression patterns of circRNA in AML were mapped through RNA-seq of clinical samples and validated in cell lines; ultimately, *circSATB1* was identified as the target for study. Subsequently, siRNA transfection was employed to investigate the cellular function, and its role in regulating premature cellular senescence in AML was corroborated. Finally, a prediction of the potential regulatory mechanism was made through bioinformatics analysis.

### 3.1. Identification of Differential circRNAs and Functional Enrichment

**Figure 1:**
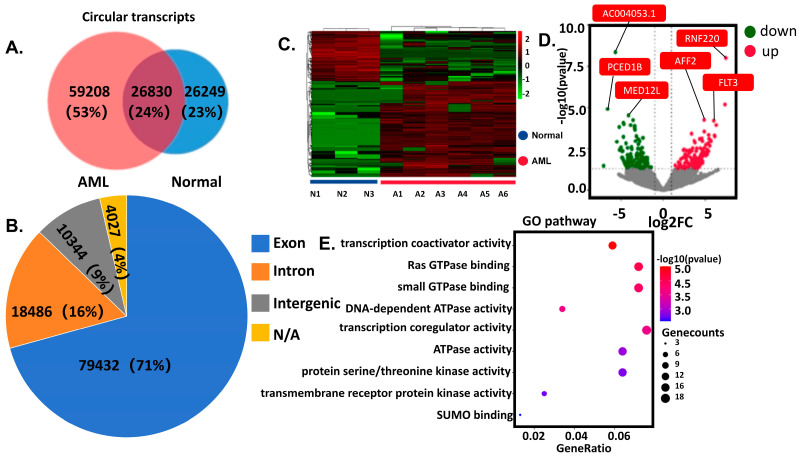
Analysis of sequencing results of circRNAs in AML. (**A**) Distribution of circRNAs in AML and healthy individuals. (**B**) Distribution of circRNAs by source. (**C**) Heat map showing differentially expressed circRNAs. (**D**) Volcano plot analysis. (**E**) Pathway enrichment analysis of differentially expressed circRNA transcripts of the parental gene. All pathways shown in the figure meet the statistical significance thresholds of *p* < 0.05, adj.*p* < 0.05, and q-value < 0.05.

For the sequencing data, a total of 112,289 circRNAs were identified through bioinformatics tools such as Find-circ, CIRI, CircRNA finder, and Circexplorer. Among these, 59,208 (53%) were unique to AML patients, approximately twice the number found in healthy volunteers (Figure 1A). Source analysis of the identified circRNAs revealed that the majority were derived from exons (79,432, 71%), followed by introns (18,486, 16%), with a small proportion originating from intergenic regions or unannotated genomic loci (Figure 1B). Subsequent differential expression analysis identified 256 up-regulated and 135 down-regulated circRNAs (Figure 1C,D). Further functional enrichment analysis of the parental genes of these differentially expressed circRNAs demonstrated significant associations with pathways such as transcription coregulator activity, Ras-GTPase activity, and DNA-dependent ATPase activity (Figure 1E). The detailed enrichment data of the GO analysis results, including *p*-values and adjusted *p*-values (adj.*p*), have been comprehensively documented in Supplementary Material Table S2. These results indicate distinct circRNAs expression patterns between AML patients and healthy volunteers.

### 3.2. circSATB1 Highly Expressed in AML Clinical Samples and Cell Lines

**Figure 2:**
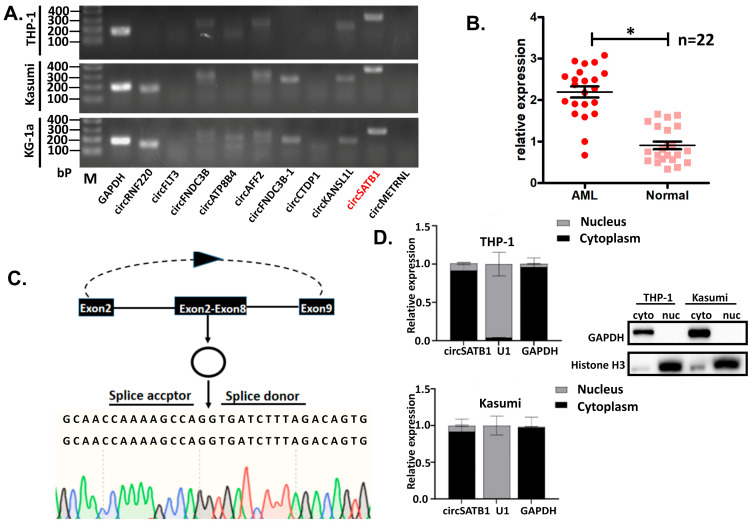
Screening and identifying *circSATB1*. (**A**) PCR analysis (gel electrophoresis) of 10 circRNAs in AML cell lines (THP-1, Kasumi, KG-1a) with GAPDH as control. (**B**) qPCR analysis of *circSATB1* expression in 22 clinical AML samples vs. matched normal controls, data presented as mean ± SD (* *p* < 0.05). (**C**) Sanger sequencing validation of *circSATB1* junction site in PCR amplification products in THP-1. (**D**) Subcellular distribution of *circSATB1* in THP-1 and Kasumi cells, identified by nuclear and cytoplasmic isolate assay. GAPDH as the cytoplasmic positive control, U1 and Histone H3 as the nuclear positive control.

Among the top ten differentially overexpressed circRNAs identified in AML (Table 1), we selected *circSATB1* for further validation based on its consistent overexpression profile across three representative AML cell lines (THP-1, Kasumi-1, and KG-1a) as determined by PCR analysis (Figure 2A). Clinical validation in the Validation Cohort using qPCR (22 matched AML-control pairs) confirmed significant up-regulation of *circSATB1* in AML specimens compared to normal controls (Figure 2B, *p* < 0.05), corroborating our initial sequencing findings and establishing *circSATB1* as a promising candidate for functional characterization.

To unequivocally confirm the circular topology of *circSATB1*, we performed Sanger sequencing of PCR-amplified products from THP-1 cell cDNA. The obtained sequences showed perfect concordance (100% identity) with the predicted back-splice junction (chr3: 18419661|18462483) in UCSC genome annotations, verifying canonical head-to-tail splicing of exons 2–9 (Figure 2C). This molecular validation confirms *circSATB1* as a bona fide circular RNA species. Subcellular fractionation experiments coupled with qPCR analysis revealed that *circSATB1* exhibited predominant cytoplasmic localization (>85% of total cellular *circSATB1*) in THP-1 and Kasumi cells, and Western blot analysis of fractionated lysates (using U1 and GAPDH as nuclear/cytoplasmic markers, respectively) confirmed the purity of subcellular fractions (Figure 2D). This distribution pattern suggests *circSATB1* may primarily function through cytoplasmic mechanisms, potentially involving post-transcriptional regulation or signaling pathway modulation.

### 3.3. circSATB1 Knockdown Diminished Cell Proliferation Without Apoptosis and DNA Damage

**Figure 3:**
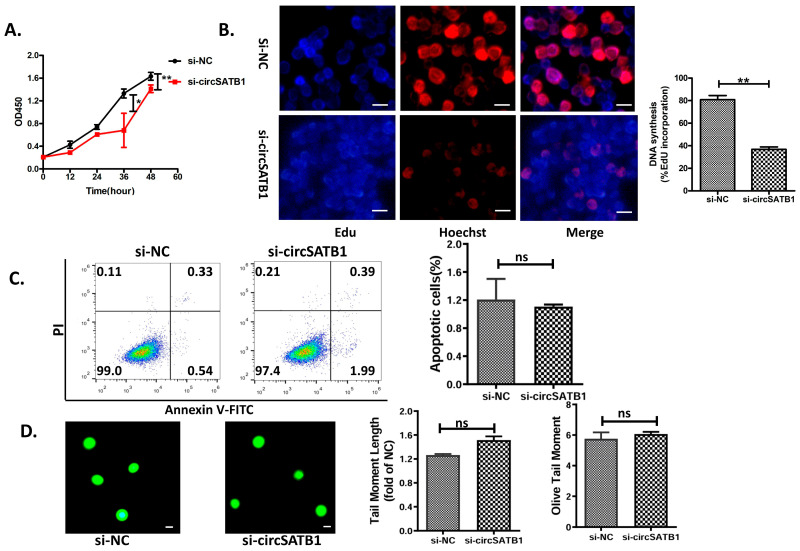
Effects of *circSATB1* knockdown on AML cell proliferation, apoptosis, and DNA integrity. (**A**) CCK-8 assays in Kasumi cells to detect cell viability in 84 h. (**B**) EdU assay in KG-1a to detect cell proliferation activity with quantitative results of DNA synthesis (right panel; *p* < 0.01 vs. si-NC). Left panel shows representative images (scale bar = 50 μm). (**C**) Flow cytometric analysis of apoptosis in THP-1 cells 48 h post-transfection. (**D**) Comet experiments in THP-1 cells, with voltage 25 V, current 300 mA, duration 30 min. n = 3 for each test. ns: no significance,* *p* < 0.05, ** *p* < 0.01 vs. si-NC.

To investigate the functional role of *circSATB1* in cellular proliferation, we designed a junction-specific siRNA targeting the back-splice junction of *circSATB1*, with scrambled siRNA (si-NC) serving as a negative control. Cellular viability was quantitatively evaluated using CCK-8 assays, and the results showed that *circSATB1* knockdown significantly inhibited the proliferation of Kasumi cell lines (Figure 3A, *p* < 0.05). In addition, EdU incorporation assays were performed in KG-1a cells at 48 h post-transfection. Quantitative fluorescence imaging demonstrated a significant reduction in EdU-positive proliferating cells following *circSATB1* knockdown compared to scramble controls (Figure 3B, 51.26% reduction in si-*cicrSATB1* group, *p* < 0.01). To assess whether the observed growth inhibition was associated with apoptosis, Annexin V-FITC/PI dual-labeling flow cytometry was conducted in THP-1 cells. As shown in Figure 3C, no significant alterations in apoptotic rates were detected. The cumulative apoptotic incidence remained below 3%, with si-*circSATB1* transfection yielding non-significant differences in early (1.99% vs. 0.54%) and late apoptotic populations (0.39% vs. 0.33%). Alkaline comet assay analysis in THP-1 cells demonstrated preserved genomic stability, with no significant differences observed in Olive Tail Moment and Tail DNA% between the *circSATB1*-knockdown and control groups (*p* > 0.05, Figure 3D). It is evident that *circSATB1* modulates AML cells through a “non-aggressive” mechanism, characterized by cytostatic proliferation arrest without inducing apoptosis or compromising DNA integrity. This aligns with the hallmark features of cellular senescence, serving as a clear indicator of the aging state in these cells.

### 3.4. circSATB1 Knockdown-Attenuated Cell Senescence

**Figure 4:**
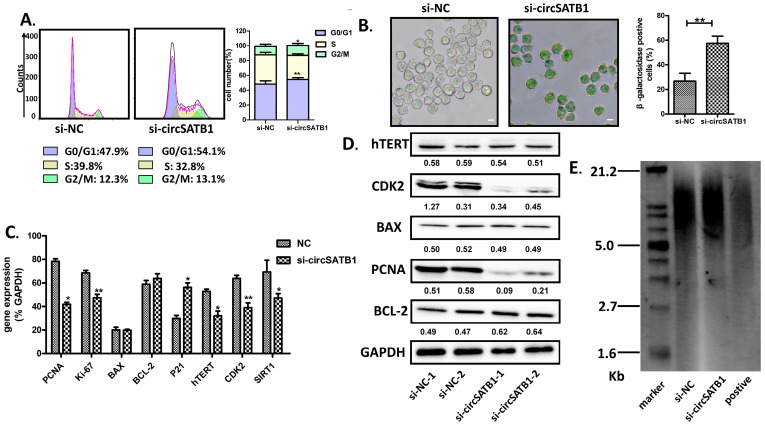
Effects of *circSATB1* knockdown on AML cell senescence. (**A**) Cell cycle analysis measured by flow cytometry in THP-1 cells. Representative histograms and quantified proportions of cells in each phase across replicates. (**B**) β-Galactosidase staining assay in THP-1. Representative images (scale bar = 50 μm) and quantification (*p* < 0.01 vs. si-NC). (**C**) qPCR and (**D**) Western blot assay to detect expression change in proliferation, apoptosis, and senescence after *circSATB1* knockdown in KG-1a, with GAPDH as control. (**E**) Telomere length analysis in KG-1a cells detected by Southern blot. n = 3 for each test. All *p*-values were calculated by *t*-test. * *p* < 0.05, ** *p* < 0.01.

To investigate the regulation of *circSATB1* on the cell cycle, flow cytometric analysis in THP-1 cells was conducted, revealing that *circSATB1* silencing induced significant cell cycle arrest. Specifically, *circSATB1*-depleted cells displayed pronounced G0/G1 phase accumulation (Figure 4A), indicative of proliferative cessation. Complementary senescence-associated β-galactosidase (SA-β-gal) staining assays confirmed the induction of cellular senescence in THP-1 cells, with a significant increase in characteristic blue staining compared to controls (Figure 4B), consistent with established senescence biomarkers. Subsequent investigation of SASP components revealed altered expression patterns of key regulatory molecules. Western blot quantification via ImageJ demonstrated significant down-regulation of proliferative markers PCNA (proliferating cell nuclear antigen), coupled with CDK2 (cyclin-dependent kinase 2) in *circSATB1*-deficient KG-1a cells (Figure 4C,D). Notably, hTERT (human telomerase reverse transcriptase) displayed discordant expression patterns across transcriptional and translational levels. While hTERT mRNA levels were significantly diminished in knockdown cells, protein expression remained statistically unaltered, suggesting potential post-transcriptional regulatory mechanisms, such as alternative splicing or translational compensation. To clarify the telomere maintenance dynamics, Southern blot analysis was employed. Quantitative assessment demonstrated comparable telomere restriction fragment lengths between control and *circSATB1*-knockdown groups in KG-1a cells (Figure 4E). These collective findings establish that *circSATB1* depletion induces acute senescence in AML cells through telomere-independent pathways, distinct from classical replicative senescence mechanisms. This phenotype aligns with emerging paradigms of stress-induced premature senescence, potentially mediated through cell cycle checkpoint activation and SASP factor modulation.

### 3.5. circSATB1 Regulatory Network Prediction

**Figure 5:**
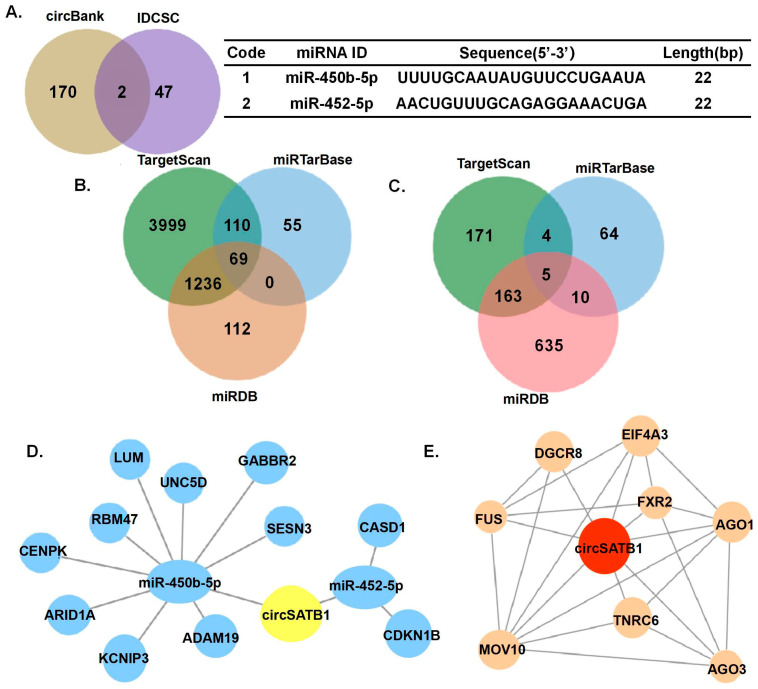
*circSATB1*-miRNA-mRNA and *circSATB1*-RBP regulatory network prediction. (**A**) CircBank and IDCSC database for *circSATB1*-bound miRNA prediction. (**B**,**C**) Venn plots show miRNA prediction for *miR-450b-5p-bound* and *miR-452-5p-bound* mRNA prediction. (**D**) *circSATB1*-miRNA-mRNA regulatory network prediction. (**E**) *circSATB1*-RBP interaction prediction.

To elucidate the potential mechanism by which *circSATB1* regulates premature cellular senescence in AML cells, we performed predictive analyses for miRNAs that may interact with *circSATB1* using the circBank and IDCSC databases. Two miRNAs, *miR-450b-5p* and *miR-452-5p*, were co-predicted by both databases (Figure 5A). Subsequently, the potential target genes of *miR-450b-5p* and *miR-452-5p* were predicted using three databases: TargetScan, miRTarBase, and miRDB, and the overlapping target genes from these databases were identified (Figure 5B,C). Based on the Score provided by miRDB, genes with a Score >90 were selected for further analysis. This yielded nine target genes for *miR-450b-5p* (*CENPK*, *GABBR2*, *RBM47*, *ARID1A*, *UNC5D*, *ADAM19*, *LUM*, *SESN3*, and *KCNIP3*) and two target genes for *miR-452-5p* (*CASD1* and *CDKN1B*) (Figure 5D).

Additionally, RNA-binding proteins (RBPs) that may interact with *circSATB1* were predicted using the catRAPID and RBPsuit databases. Eight RBPs were co-predicted: AGO1, AGO3, DGCR8, EIF4A3, FUS, FXR2, MOV10, and TNRC6. The protein–protein interaction (PPI) network of these RBPs was analyzed using the STRING database, and experimentally validated interactions (as documented in STRING) were visualized (Figure 5E).

**Figure 6:**
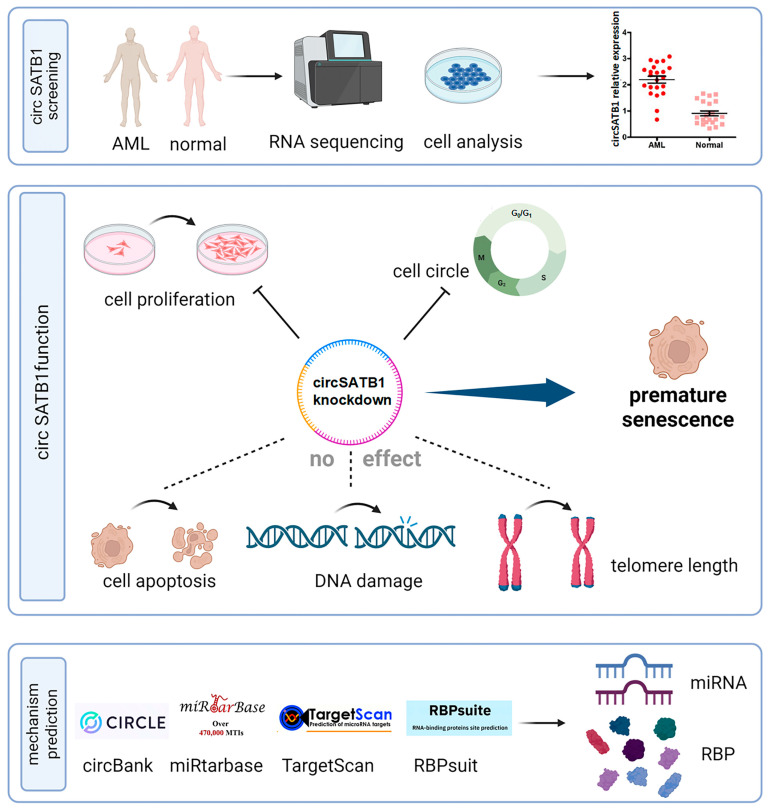
Schematic characterization of the research workflow, functional characterization, and mechanistic prediction of *circSATB1* in AML.

These results demonstrate that *circSATB1* is associated with cellular senescence, exhibits high expression in AML, and exerts an anti-senescence effect. Knockdown of *circSATB1* promotes premature senescence and inhibits cell proliferation (Figure 6), suggesting its potential as a critical therapeutic target for inducing AML cell senescence.

## 4. Discussion

*circSATB1*, a previously uncharacterized circular RNA, was highly expressed in elderly AML patients and orchestrates a telomere-independent senescence program upon knockdown. Using multi-omics and functional validation, we demonstrate that (1) *circSATB1* is significantly overexpressed in AML clinical samples and cell lines (Figure 2A,B); (2) its inhibition induces G0/G1 arrest and premature senescence without triggering apoptosis or DNA damage (Figure 3 and Figure 4); (3) this senescence phenotype occurs independently of telomere shortening (Figure 4E). These results establish *circSATB1* as a novel regulator of cellular senescence in AML.

Population aging is a global public health challenge. Age-related diseases are becoming an increasingly serious public health concern [15]. It is both imperative and urgent to address these challenges through specific aging biomarkers. Also, their impact on age-related diseases is relatively unexplored, and their potential mechanisms to minimize or alleviate this rapid aging trend remain unclear. Recent research has proposed twelve hallmarks of aging, such as genomic instability, telomere attrition, mitochondrial dysfunction, cellular senescence, and chronic inflammation, which all contribute to understanding the progression of aging. Identifying biomarkers of aging and risks associated with age-related diseases is crucial for supporting public health initiatives for healthy aging, developing a priority control list, and enhancing regulation. Acute myeloid leukemia, the most common adult leukemia, predominantly affects individuals aged 60–70 years [16]. Elderly patients (≥65 years) exhibit significantly higher mortality rates, with a dismal 5-year relative survival rate of only 29.5% [17].

circRNAs play important roles in a variety of tumors. Signature circRNAs have been identified in Alzheimer’s disease [18], brain cell glioma [19], lung cancer [20], and other tumors in the elderly. In addition, tissue fluids and body fluids (saliva, blood, etc.) have been found to be rich in circRNAs [21], which are ideal biomarkers for liquid biopsy and may be a potential target for hematological tumor therapy, but it is not yet clear which circRNAs are involved in the development of AML. Certain circRNAs exhibit spatiotemporal expression patterns correlating with cell cycle phases, while others serve as molecular indicators of cellular senescence. As the third tumor barrier following apoptosis and DNA damage repair, senescence induces irreversible growth arrest while maintaining metabolic activity, thereby limiting cancer proliferation [22]. The Belgian group’s “one-two punch” strategy “CDC7 inhibitor-induced senescence” + “mTOR blocker-triggered apoptosis” effectively eliminated hepatocellular carcinoma cells with improved patient tolerance compared to conventional cytotoxic therapies [23]. Achieving cancer eradication while preserving quality of life remains a therapeutic dilemma, particularly for elderly AML patients. Therefore, exploring a similar therapeutic strategy to be adopted for AML is of significant interest. Identifying circRNAs that induce senescence in AML cells is a crucial step in this direction. Our study addresses this gap by identifying *cirSATB1* as a senescence modulator in AML. While our data demonstrate that *circSATB1* knockdown induces senescence (Figure 3 and Figure 4), the preliminary nature of the predicted regulatory networks (Figure 5) must be emphasized. Causal relationships between *circSATB1* and senescence effectors remain to be established.

The ability of cancer cells to evade senescence is contingent on their sustained proliferative capacity. Cellular senescence can be classified into replicative senescence (telomere-dependent) and premature senescence (telomere-independent and stress-induced) [24,25,26,27,28]. In this study, we demonstrated that *circSATB1* knockdown induced premature senescence in AML cells, characterized by maintenance of telomere length and obstruction of cell cycle progression. This suppression of uncontrolled AML cell proliferation offers a novel and mild therapeutic approach, providing a framework for identifying more tolerable AML treatment options. Such options include the induction of cellular senescence to halt disease progression, which holds significant prognostic value for elderly patients. Our findings align with emerging interest in senescence-inducing therapies [12,14,22,23], though the specific role of circRNAs in AML senescence was previously unexplored. The predicted interaction networks involving *miR-450b-5p*, *miR-452-5p*, and specific RBPs (Figure 5) provide testable hypotheses for future mechanistic studies to understand how *circSATB1* exerts its anti-senescence function. While our data provided evidence for identifying *circSATB1* as a novel regulator of senescence in AML and suggest its potential as a therapeutic target, several limitations warrant acknowledgment: (1). Cohort size: Our conclusions are derived from in vitro analyses using AML cells from a limited cohort, which may not fully capture the heterogeneity of AML. Larger-scale validation in diverse populations is needed. (2). Model constraints: The exclusive use of in vitro models precludes assessment of systemic factors. Future work should integrate probing physiological relevance. (3). Mechanistic depth: Although we identified *circSATB1* as a novel regulator of senescence in AML and suggest its potential as a therapeutic target, causal links require further deconvolution. Its therapeutic potential and mechanism of action remains untested.

## 5. Conclusions

In this study, we obtained 391 circRNAs significantly differentially expressed in AML by bioinformatics, which are potentially valuable in the study of AML. Experimental means were screened to obtain *circSATB1*, which is significantly highly expressed in AML and stably expressed in AML cells, and its circRNA properties were verified, and it was found to be mainly present in the cytoplasm and expressed in a variety of cancers. By knockdown of *circSATB1* in AML cells, it was found that its knockdown could inhibit cell proliferation and promote premature cell senescence, while it had no significant effect on apoptosis and DNA damage. Preliminary prediction of the mechanism of action network of *circSATB1* by bioinformatics means yielded two miRNAs and eight RBPs that might bind to *circSATB1*. These findings identify *circSATB1* as a novel regulator of senescence in AML and suggest its potential as a therapeutic target. Future studies are warranted to validate the predicted regulatory networks and assess the therapeutic efficacy of targeting *circSATB1* in vivo.

## Figures and Tables

**Table 1:** Top 10 circRNAs highly expressed in AML.

Genomic Coordinates	Maternal Gene	Typology	Lengths (bp)
chr1:44877652|44878394	*RNF220*	exon	742
chr13:28598997|28602425	*FLT3*	exon	348
chr3:171969049|172028671	*FNDC3B*	exon	746
chr15:50330964|50366382	*ATP8B4*	exon	334
chrX:147733519|147744289	*AFF2*	exon	982
chr3:171969049|172025291	*FNDC3B*	exon	692
chr18:77455224|77464917	*CTDP1*	exon	458
chr2:211018218|211019335	*KANSL1L*	exon	1117
chr3:18419661|18462483	*SATB1*	exon	1599
chr17:81042813|81043199	*METRNL*	exon	386

## Data Availability

The data presented in this study are available upon request from the corresponding author. The RNA sequencing datasets have been deposited to the NCBI’s Gene Expression Omnibus GEO database and are accessible through GEO Series accession numbers GSE229582 (https://www.ncbi.nlm.nih.gov/geo/query/acc.cgi?acc=GSE229582, accessed on 11 January 2025).

## Supplementary Materials

The following supporting information can be downloaded at: https://www.mdpi.com/article/10.3390/cells14151181/s1, Table S1: Demographic and clinical characteristics of RNA sequencing donors; Table S2: Statistical results of GO enrichment analysis.

## Abbreviations

The following abbreviations are used in this manuscript:

AML	Acute myeloid leukemia
CR	complete remission
OS	overall survival
AD	Alzheimer’s disease
BMSC	bone marrow mesenchymal stem cells
OP	osteoporosis
ASOs	Antisense oligonucleotides
TIS	Therapy-induced senescence
GBM	glioblastoma
RBP	RNA-binding proteins
qPCR	Quantitative PCR (polymerase chain reaction)
PI	Propidium Iodide
EdU	5-Ethynyl-2′-deoxyuridine
SASP	senescence-associated secretory phenotype
TRF	terminal restriction fragment
