# Supplementary material for: circSATB1 Modulates Cell Senescence in Age-Related Acute Myeloid Leukemia: A Mechanistic Proposal
Source: Cells. 2025 Jul 31;14(15):1181. doi: 10.3390/cells14151181 (PMC12346576; doi:10.3390/cells14151181)
Supplement: Supplementary file 1 [file cells-14-01181-s001.zip › Table S1.pdf]

Demographic and Clinical Characteristics of RNA Sequencing Donors

Sample ID	FAB classification	Gender	Age	Cytogenetics	Molecular biology	Risk stratification
01	M4a	F	51	46,XX	EV11 mutation	Intermediate risk
02	M2b	F	50	46,XX;t(8;21)(q22; q22)	AML1/ETO	Low risk
03	M1	M	53	46,XY	NPM1 mutation	Low risk
04	M2a	F	51	46,XX	FLT3-ITD mutation	High risk
05	M5a	M	60	46,XY	FLT3-ITD mutation	High risk
06	M2a	M	52	46,XY	CEBPA mutation	Intermediate risk
07		F	55	46,XX		
08		M	62	46,XY		
09		F	61	46,XX		
