# Supplementary material for: circSATB1 Modulates Cell Senescence in Age-Related Acute Myeloid Leukemia: A Mechanistic Proposal
Source: Cells. 2025 Jul 31;14(15):1181. doi: 10.3390/cells14151181 (PMC12346576; doi:10.3390/cells14151181)
Supplement: Supplementary file 1 [file cells-14-01181-s001.zip › Table S2.pdf]

Table S2: Statistical Enrichment of Functional GO Terms

Description	GeneRatio	BgRatio	pvalue	p.adjust	qvalue	Count
transcription coactivator activity	14/236	267/18352	9.85E-06	0.004638006	0.004353475	14
Ras GTPase binding	17/236	415/18352	2.82E-05	0.00652403	0.006123796	17
small GTPase binding	17/236	428/18352	4.16E-05	0.00652403	0.006123796	17
DNA-dependent ATPase activity	8/236	108/18352	7.74E-05	0.007850435	0.007368829	8
transcription coregulator activity	18/236	498/18352	8.33E-05	0.007850435	0.007368829	18
ATPase activity	15/236	423/18352	0.000399191	0.031336487	0.029414068	15
protein serine/threonine kinase activity	15/236	435/18352	0.000533932	0.033955025	0.031871964	15
transmembrane receptor protein kinase activity	6/236	80/18352	0.000576731	0.033955025	0.031871964	6
SUMO binding	3/236	15/18352	0.000852164	0.044596579	0.041860684	3
